# Intermittent fasting during adjuvant chemotherapy may promote differential stress resistance in breast cancer patients

**DOI:** 10.1186/s43046-022-00141-4

**Published:** 2022-09-12

**Authors:** Enas M. Omar, Gamal A. Omran, Mohamed F. Mustafa, Noha M. El-Khodary

**Affiliations:** 1 Damanhour Oncology Center, Damanhour, 22511 Egypt; 2 Department of Biochemistry, Faculty of Pharmacy, Damanhour University, Damanhour, 22511 Egypt; 3 Department of Clinical Oncology, Faculty of Medicine, Alexandria University, Alexandria, 21500 Egypt; 4 Department of Clinical Pharmacy, Faculty of Pharmacy, Kafrelsheikh University, Kafrelsheikh, 33511 Egypt

**Keywords:** Intermittent fasting, Chemotherapy, Breast cancer, Differential stress resistance, Toxicity, Insulin, Glucose, Vomiting

## Abstract

**Background:**

Preclinical studies prove that short-term fasting secures healthy cells against chemotherapy side effects and makes malignant cells more vulnerable to them. This study aimed to examine the effects of intermittent fasting (IF) during adjuvant chemotherapy AC (doxorubicin, cyclophosphamide) protocol in breast cancer (BC) patients.

**Methods:**

Forty-eight newly diagnosed human epidermal growth factor receptor 2-negative (HER2 negative) BC patients were divided equally into two groups (24 each). The first group was recruited to fast intermittently for three consecutive days around chemotherapy for 18 h a day from 12 am to 6 pm and eats through 6 h a day from 6 pm to 12 am with permission of drinking water during fasting hours (IF group). This IF was repeated every 3 weeks for four cycles. The second group is a non-fasting (NF) group that was allowed to eat regularly. Toxicity in the two groups was compared. Hematologic, metabolic, and inflammatory parameters were measured and compared.

**Results:**

Toxicity related to the gastrointestinal tract (GIT) was reduced in the IF group. Hematologic parameters showed no significant variations between the two studied groups after cycle 4. There was a significant increase in median glucose and median insulin levels (*P* < 0.001 and *P* = 0.001, respectively) in the NF group between baseline and after cycle 4. In addition, there was a significant decrease in the median insulin level (*P* = 0.002) in the IF group between the two time points.

**Conclusion:**

IF throughout chemotherapy was well tolerated and decreased the toxicity of chemotherapy. Additionally, IF-improved metabolic profiles of patients may have a positive impact on the clinical efficacy of chemotherapy.

**Supplementary Information:**

The online version contains supplementary material available at 10.1186/s43046-022-00141-4.

## Background

Breast cancer is the most commonly diagnosed cancer and the major cause of cancer-related death among women worldwide [1]. Although cancer treatments such as surgery, chemotherapy, and radiation therapy have demonstrated significant therapeutic efficacy, damage to normal tissue and the resulting side effects are unavoidable, and further prognostic improvement remains a challenge. As a result, in addition to established treatment techniques, other aids must be identified in order to improve prognosis [2]. The correlation between dietary changes and their effects on cancer incidence and treatment has increased over the last decade [3]. Investigations of long-term calorie-confined human subjects have demonstrated a decrease in metabolic and hormonal factors associated with cancer hazards [4]. Calorie restriction can be achieved through overall dietary reduction or by intermittent fasting (IF) [5]. IF is a nutritional strategy that requires fasting for varied periods, typically for 12 h or longer [6]. Fasting as a medicinal aid has been reported to be a reproducible and effective intervention strategy for protecting mammals from tumors and prolonging overall survival [2]. In human subjects, fasting for 36 h was safe and well tolerated [7]. A case series of 10 patients with varied types of cancer demonstrated that fasting in combination with chemotherapy was feasible and might reduce chemotherapy-induced side effects [8]. The effects of short-term fasting on sensitivity to chemotherapy cause differences between healthy somatic and cancer cells, which is a phenomenon known as differential stress resistance (DSR) [9]. Healthy somatic cells enter a self-maintenance stage in response to fasting. This happens since fasting decreases the levels of circulating hormones and metabolites within the body, such as glucose, insulin, and insulin-like growth factor-1 (IGF-1). Nutrient deprivation signals healthy cells to regulate cell division and growth, thereby protecting them from chemotherapy, whereas the mutations in cancer cells make them less capable of adaptation to extraordinary situations created during fasting, and therefore, fasting cycles render tumor cells more sensitive to chemotherapy [10]. Consequently, IF is a promising strategy for increasing chemotherapy effectiveness and tolerability.

IGF-1 has been shown to be one of the significant growth factors that encourages cell division and proliferation. IGF-binding proteins regulate serum IGF-I concentrations. IGF-1 is significantly reduced by fasting, while its binding proteins show dissimilar patterns of progress accordingly in response to fasting. IGF-binding protein 1 (IGFBP-1) increases rapidly while fasting overnight and is easily suppressed by calorie intake [11, 12].

It has been demonstrated that short-term fasting causes a decrease in circulating IGF-1 as well as an increase in IGFBP-1 level, which is associated with increased stress resistance [13].

When compared to an eating pattern in which food is ingested over long periods of time (typically 12 h or more daily), IF eating habits may offer a wide range of health benefits in humans, including improved glucose metabolism [14], lower inflammation [15], and better cell tolerance to stress and disease [16]. These effects have been proven in animal research, although some of the effects have yet to be proven in humans [17].

The aim of this study was to determine the safety and practicality of IF during chemotherapy, as well as its ability to promote DSR according to the metabolic changes induced in BC patients.

## Methods

### Patients

This was a randomized controlled clinical trial conducted on 48 patients recently diagnosed HER2-negative BC patients aged from 27 to 66 years who were recruited from the Damanhour Oncology Center during the period between February 2019 and January 2020. Approval was obtained from the Damanhour Oncology Center’s Ethics Committee and Research Ethics Committees of Damanhour University’s Faculty of Pharmacy ref. no. 618PP2) in accordance with the Declaration of Helsinki 1975, revised in Hong Kong in 1989. Prior to participating in the study, all patients signed written informed consent.

### The criteria for inclusion

Patients with breast cancer were aged ≥ 18 years with adequate bone marrow function (i.e., white blood counts > 3.0 × 10^9^/l, absolute neutrophil count ≥ 1.5 × 10^9^/l, and platelet count ≥ 100 × 10^9^/l), adequate cardiac function (i.e., left ventricular ejection fraction (LVEF) ≥ lower limit of normal (LLN)), adequate renal function (i.e., calculated creatinine clearance ≥ 50 ml/min), adequate liver function (i.e., bilirubin ≤ 1.5 × upper normal limit (UNL) range, alanine aminotransferase (ALT) and/or aspartate aminotransferase (AST) ≤ 2.5 × UNL, alkaline phosphatase ≤ 5 × UNL), and HER-2-negative patients.

### The criteria for exclusion

The criteria were diabetes mellitus, pregnancy, current lactation, having a low body mass index (BMI) < 20.5, or had lost more than 10% of their weight in the preceding year.

### Study design

Patients were randomized in a 1:1 ratio into two groups (24 patients each) using permuted block randomization according to a manually generated random sequence to enroll either in the intermittent fasting (IF) group or in the non-fasting (NF) group. The first group was IF group that was recruited to fast intermittently for three consecutive days: the day before, during, and the day after chemotherapy. The second group was NF group that was allowed to eat regularly.

The fasting was designed to be for 18 h from 12 am to 6 pm, with eating through 6 h from 6 pm to 12 am and with permission to drink water during fasting hours and eat small quantities of food consisting mainly of vegetables, fruits, and a small amount of proteins and carbohydrates with a limitation of sugar and fats. The total quantity of calories consumed per day during the 6 h of eating should not exceed 750 kcal, and this amount of calories was calculated with the assistance of a nutrition specialist and was supplied to the fasting group patients to follow and adhere to as shown in a supplementary file (Supplement 1). This IF was repeated every 3 weeks for four cycles, with the sugar and fat limitations being extended on all four cycles. Patients were considered compliant when they completed the fasting regimen through all over the 4 cycles of chemotherapy.

All patients were encouraged to follow a balanced eating pattern that includes lots of fruits and vegetables; moderate amounts of whole grains; plant protein sources such as nuts, beans, and lentils; small servings of fish, poultry, and lean meats; and nonfat or low-fat dairy foods.

### Drugs

Patients received doxorubicin + cyclophosphamide (AC) chemotherapy consisting of doxorubicin 60 mg/m^2^ IV infusion for 15 min + cyclophosphamide 600 mg/m^2^ IV infusion for 1/2 h, and the cycles were repeated every 3 weeks for 4 cycles. Granisetron (serotonin 5-HT_3_ receptor antagonist; 1 mg) was given as an antiemetic agent and dexamethasone 8 mg to prevent hypersensitivity reactions and fluid retention prior to chemotherapy infusions. All patients received the same dose of steroids.

### Blood sampling

Two-hour postprandial blood samples were collected in the morning from 9:00 am to 11:00 am 2 days prior to chemotherapy (baseline) and at the end of four cycles of the AC protocol 8 days after the 4th chemotherapy cycle. The effects of IF were evaluated by recording (1) metabolic parameters (insulin, glucose, IGF-1, and IGFBP1); (2) hematologic parameters (erythrocyte, platelet, and leukocyte count); and (3) inflammatory marker (C-reactive protein (CRP). For measurement of metabolic parameters, blood samples were drawn in serum-separating tubes, allowed 20 min for coagulation, and then centrifuged at 4000 rpm for 10 min. Serum was separated and stored at − 80 °C until analysis. Freezing of serum samples at − 80 °C was available at the Damanhour University’s Faculty of Pharmacy. Human insulin levels were measured by an enzyme-linked immunosorbent assay (ELISA) kit (Perfect Ease Biotech (Beijing) Co., Ltd., USA). Human insulin-like growth factor-1 (IGF-1) and human insulin-like growth factors binding protein 1 (IGFBP-1) were measured by ELISA kits (Sun Red Company, China). CRP was measured by a latex-enhanced turbidimetry immunoassay (Omega Diagnostic Ltd., Scotland, UK). For measurement of hematologic parameters, blood samples were collected in EDTA tubes and analyzed by the automated hematology analyzer.

### Toxicity

Throughout each cycle, patients were asked to describe mild, moderate, or severe unpleasant effects, including fatigue, headache, diarrhea, constipation, nausea, vomiting, hair loss, and mouth sores. The Common Terminology Criteria for Adverse Events version 5 (CTCAE v.5) [18] were used to grade the adverse effects and hematological toxicity reported by the Damanhour Oncology Center’s authorized clinical laboratory.

Unacceptable fasting-related toxicity was defined as patients were being hospitalized during the fasting period (for reasons that did not seem to be attributed to disease, chemotherapy, or postoperative complications).

### Statistical analysis

Based on previously treated trial cases [19] (IGF-1), we conducted a power analysis (G*Power version 3.1 statistical software, Franz Faul, Universität Kiel, Germany). To estimate the required sample size given, power, and effect size, a difference between two independent means (two groups) analyses was used. The input parameters were *α* error probability of 0.05, an effect size (f) of 0.98, and a power of 0.95, and the number of groups was 2. The findings indicated a minimum sample size of *n* = 48 samples (24 samples for each group).

All statistical tests were conducted using the IBM SPSS programming bundle version 20.0 (IBM Corporation, Armonk, NY). Numbers and percent were used to describe subjective data. The Kolmogorov–Smirnov test was used to ensure that the distribution was normal. The range (minimum and maximum), mean, standard deviation, and median were used to represent quantitative data. An estimate of *P* < 0.05 was considered statistically significant. The tests used were the chi-square test for categorical variables to compare between groups and the Monte Carlo adjustment for chi-square when more than 20% of the cells had an anticipated count of less than 5. To compare the two groups, the Student *t*-test for normally distributed quantitative data and the Mann–Whitney test for abnormally distributed quantitative variables were used. The Wilcoxon signed-rank test for abnormally distributed quantitative data and the paired *t*-test for regularly distributed quantitative values of the variables were used to compare the two periods.

## Results

### Patient characteristics

**Fig. 1:**
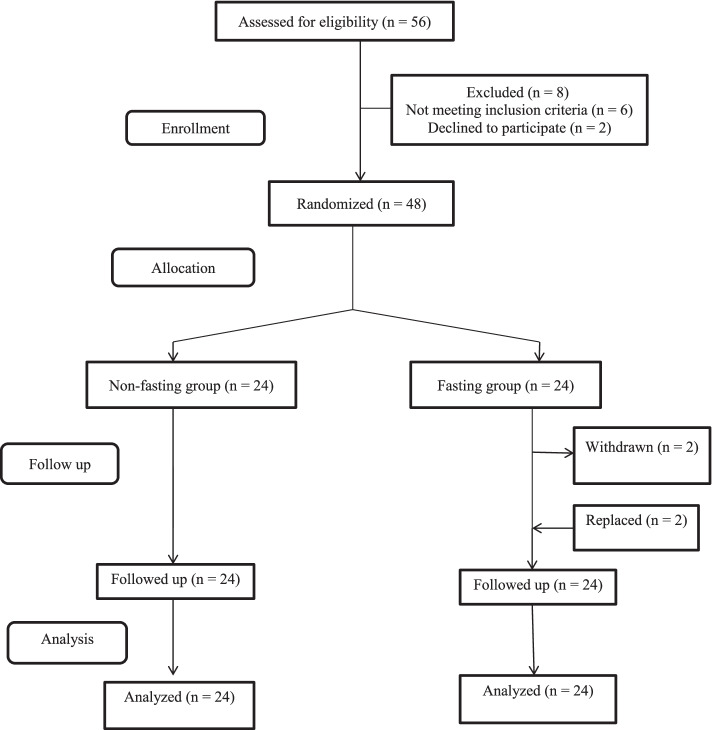
The study flow diagram

Initially, 56 patients were included in this study. After checking the inclusion and exclusion criteria, as previously described for the selection of patients, 48 patients were maintained throughout the study. However, two patients in the IF group withdrew from fasting after the second chemotherapy cycle due to their desire not to continue and were then replaced. The study flowchart is shown in Fig. 1.

The average age of the study participants was 43.96 ± 7.37 and 47.42 ± 11.73 years for the IF and NF groups, respectively. The demographic and baseline biological characteristics of the participants were summarized in Table 1.

**Table 1:** Comparison between the two studied groups according to baseline patient characteristics

	**Intermittent fasting** **(*n* = 24)**	**Non-fasting (*n* = 24)**	***P***
**Age (years)**			
Mean ± SD	43.96 ± 7.37	47.42 ± 11.73	0.229
Median (min.–max.)	44.0 (30.0–60.0)	45.0 (27.0–66.0)	
**Weight (kg)**			
Mean ± SD	88.83 ± 14.14	87.83 ± 17.40	0.828
Median (min.–max.)	86.0 (65.0–113.0)	86.50 (49.0–130.0)	
**Height (m)**			
Mean ± SD	1.67 ± 0.05	1.66 ± 0.05	0.330
Median (min.–max.)	1.68 (1.55–1.75)	1.66 (1.50–1.72)	
**BMI (kg/m**^**2**^**)**			
Mean ± SD	31.77 ± 4.06	31.89 ± 5.35	0.933
Median (min.–max.)	32.23 (23.88–36.96)	32.33 (21.67–43.94)	
**ER**	**No. %**	**No. %**	^MC^*p* = 0.462
Negative	6 (25.0%)	4 (16.7%)	
1 +	0 (0.0%)	1 (4.2%)	
2 +	8 (33.3%)	5 (20.8%)	
3 +	10 (41.7%)	14 (58.3%)	
**PR**			
Negative	7 (29.2%)	4 (16.7%)	^MC^*p* = 0.595
1 +	3 (12.5%)	3 (12.5%)	
2 +	3 (12.5%)	6 (25.0%)	
3 +	11 (45.8%)	11 (45.8%)	
**Grade**			
I	0 (0.0%)	4 (16.7%)	^MC^*p* = 0.141
II	18 (75.0%)	16 (66.7%)	
III	6 (25.0%)	4 (16.7%)	
**Stage**			
IA	4 (16.7%)	6 (25.0%)	^MC^*p* = 0.236
IIA	12 (50.0%)	5 (20.8%)	
IIB	2 (8.3%)	4 (16.7%)	
IIIA	1 (4.2%)	4 (16.7%)	
IIIC	5 (20.8%)	5 (20.8%)	

*SD *standard deviation, *BMI* body mass index, *ER *estrogen receptor, *PR *progesterone receptor, *MC* Monte Carlo

*P P*-value for comparing between intermittent fasting and non-fasting

^*^Statistically significant at *P* < 0.05

Both groups had comparable characteristics, including age, BMI, estrogen and progesterone receptors, grading, and stage of cancer.

All patients finished 4 cycles of AC protocol. There were no significant variations in chemotherapy-related modifications between the two groups.

### Toxicity

The most noticeable toxicity grades were grades 1 and 2. There was just a single patient who experienced grade 3 neutropenia in the NF group. No patient experienced grade 4 or 5 toxicity during the chemotherapy in either group. The most frequently observed side effects and percentages of occurrence and grading were recorded in Table 2. No significant difference was observed between the two patient groups in the incidence of thrombocytosis, neutropenia, headache, hair loss, or fatigue. None in the IF group experienced mouth sores or diarrhea compared to 8 of 24 (33.3%) and 12 of 24 (50%) patients in the NF group (*P* = 0.004 and *P* < 0.001, respectively). A total of 2/24 (8.3%) patients experienced vomiting, and 17/24 (70.8%) patients experienced nausea in the IF group, compared to 19/24 (79.2%) patients who experienced vomiting and 24/24 (100%) patients experienced nausea in the NF group (*P* < 0.001 and *P* = 0.009, respectively). None in the NF group suffered from constipation, compared to 8/24 (33.3%) patients in the IF group (*P* = 0.004).

**Table 2:** Grading of toxicity during the 4 cycles in the two studied groups

**Toxicity**	**Intermittent fasting** **(*n* = 24)**			**Non-fasting (*n* = 24)**			***P***
	Grade 1 **No. %**	Grade 2 **No. %**	Grade 3 **No. %**	Grade 1 **No. %**	Grade 2 **No. %**	Grade 3 **No. %**	
**Thrombocytosis**	6 (25%)	-	-	7 (29.167%)	1 (4.167%)	^−^	0.525
**Neutropenia**	2 (8.33%)	2 (8.33%)	-	-	2 (8.33%)	^1 (4.17%)^	^FE^*p* = 1.000
**Headache**	7 (29.17%)	2 (8.33%)	-	5 (20.83%)	1 (4.167%)	^−^	0.350
**Mouth sores**	-	-	-	5 (20.83%)	3 (12.5%)	^−^	^FE^*p* = 0.004**
**Diarrhea**	-	-	-	9 (37.5%)	3 (12.5%)	^−^	< 0.001***
**Constipation**	6 (25%)	2 (8.33%)	-	-	-	^−^	^FE^*p* = 0.004**
**Vomiting**	2 (8.33%)	-	-	14 (58.3%)	5 (20.83%)	^−^	< 0.001***
**Nausea**	16 (66.67%)	1 (4.17%)	-	19 (79.16%)	5 (20.83%)	^−^	^FE^*p* = 0.009**
**Hair loss**	12 (50%)	6 (25%)	-	19 (79.16%)	4 (16.67%)	^−^	^FE^*p* = 0.188
**Fatigue**	6 (25%)	3 (12.5%)	-	11 (45.83%)	2 (8.33%)	^−^	0.247

*FE* Fisher exact. *P P*-value for comparing between intermittent fasting and non-fasting group

^*^P < 0.05

^**^P < 0.01

^***^P < 0.001

### Hematologic parameters

Hemoglobin levels and red blood cell counts were significantly decreased, while platelet counts were significantly increased after cycle 4 compared to baseline in both groups. IF group showed a significant decrease in white blood cells and neutrophil counts after cycle 4 compared to baseline, as shown in Table 3. There were no significant differences in hematologic parameters between the two studied groups after cycle 4, as shown in Table 4.

**Table 3:** Comparison between the two studied periods according to hematologic parameters in each group

Hematologic parameters	Intermittent fasting (*n* = 24)			Non-fasting (*n* = 24)		
	**Baseline**	**After cycle 4**	***P***	**Baseline**	**After cycle 4**	***P***
**RBC (× 10**^**6**^**/µl)**	4.89 ± 0.45	4.61 ± 0.53	(0.004**)	4.99 ± 0.45	4.61 ± 0.72	(0.016*)
**HG (g/dl)**	12.35 ± 1.45	11.65 ± 1.27	(0.001**)	12.36 ± 1.19	11.89 ± 1.30	(0.002**)
**PLT (× 10**^**3**^**/µl)**	317.5 ± 73.23	383.6 ± 79.85	(< 0.001***)	309.71 ± 112.41	376.04 ± 119.97	(0.009**)
**WBC (× 10**^**3**^**/µl)**	8.33 ± 2.92	5.87 ± 1.87	(< 0.001***)	6.54 ± 1.44	5.98 ± 2.33	(0.152)
**NEUT (× 10**^**3**^**/mm**^**3**^**)**	5.08 ± 2.82	3.45 ± 1.46	(< 0.001***)	3.54 ± 1.07	3.76 ± 1.65	(0.493)

*RBC* red blood cells, *HG* hemoglobin, *PLT* platelets, *WBC* white blood cells, *NEUT*, neutrophils

Data presented as mean ± SD. *P P*-value for comparing between baseline and after cycle 4 in each group

^*^P < 0.05

^**^P < 0.01

^***^P < 0.001

**Table 4:** Comparison between the two studied groups according to hematologic parameters after cycle 4

Hematologic parameters	Intermittent fasting (*n* = 24)	Non-fasting (*n* = 24)	*P*
**RBC (× 10**^**6**^**/µl)**	4.61 ± 0.53	4.61 ± 0.72	0.982
**HG (g/dl)**	11.65 ± 1.27	11.89 ± 1.30	0.526
**PLT (× 10**^**3**^**/µl)**	383.6 ± 79.85	376.04 ± 119.97	0.799
**WBC (× 10**^**3**^**/µl)**	5.87 ± 1.87	5.98 ± 2.33	0.864
**NEUT (× 10**^**3**^**/mm**^**3**^**)**	3.45 ± 1.46	3.76 ± 1.65	0.415

*RBC* red blood cells, *HG* hemoglobin, *PLT* platelets, *WBC* white blood cells, *NEUT *neutrophils

Data presented as mean ± SD. *P P*-value for comparing between intermittent fasting group and non-fasting group

### Metabolic and inflammatory parameters

Between baseline and cycle 4, there was no significant difference in median glucose levels in the IF group, while there was a significant increase in median glucose and median insulin levels in the NF group (*P* < 0.001 and *P* = 0.001, respectively). On the other hand, the median insulin level in the IF group decreased significantly (*P* = 0.002) between the two time points. IGF-1 and IGF-BP1 levels in both groups did not vary significantly over time. Between the two time points, median CRP levels in the NF group were substantially higher (*P* < 0.001) than those in the IF group (Table 5).

**Table 5:** Comparison between the two studied periods according to metabolic and inflammatory parameters in each group

Metabolic parameters	Intermittent fasting			Non-fasting		
	**Baseline**	**After cycle 4**	***P***	**Baseline**	**After cycle 4**	***P***
**Glucose (mg/dl)**	**(*n* = 24)**	**(*n* = 24)**		**(*n* = 24)**	**(*n* = 24)**	
Median (min.–max.)	99.50 (72.0–141.6)	98.50 (77.0–123.0)	(0.516)	105.0 (82.0–161.0)	131.8 (84.0–251.0)	(< 0.001***)
**Insulin (µIU/ml)**	**(*n* = 21)**	**(*n* = 21)**		**(*n* = 20)**	**(*n* = 20)**	
Median (min.–max.)	61.73 (0.0–210.0)	12.23 (0.0–165.7)	(0.002**)	37.85 (0.0–210.0)	90.42 (0.0–210.0	(0.001**)
**IGF1 (ng/ml)**	**(*n* = 21)**	**(*n* = 21)**		**(*n* = 20)**	**(*n* = 20)**	
Median (min.–max.)	13.48 (2.23–50.33)	9.15 (0.0–49.85)	(0.085)	8.61 (0.0–50.33)	9.90 (0.0–39.75)	(0.411)
**IGFBP1 (ng/ml)**	**(*n* = 19)**	**(*n* = 20)**		**(*n* = 14)**	**(*n* = 17)**	
Median (min.–max.)	347.6 (0.0–2082.0)	274.5 (0.0–2060.3)	(0.372)	297.7 (0.0–2221.9)	340.3 (0.0 – 1856.5)	(0.507)
**CRP (mg/L)**	**(*n* = 22)**	**(*n* = 22)**		**(*n* = 19)**	**(*n* = 19)**	
Median (min.–max.)	4.50 (1.80–7.10)	4.10 (2.10–10.0)	(0.445)	3.50 (3.0–6.20)	4.70 (3.40–10.50)	(< 0.001***)

*IGF1* insulin-like growth factor-1, *IGFBP1* IGFbinding protein 1, *CRP* C-reactive protein

*P*: *P* value for comparing between baseline and after cycle 4 in each group

^*^P < 0.05

**P < 0.01

***P < 0.001

**Fig. 2:**
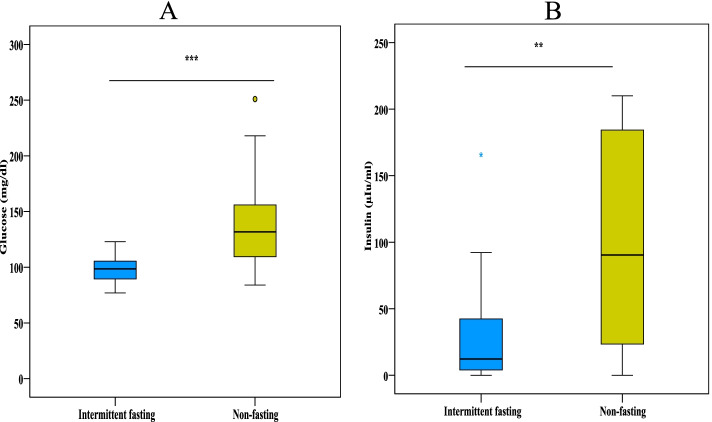
A comparison between the two examined groups concerning glucose (mg/dl) and insulin (µIU/ml) levels after cycle 4. **A** shows a significant increase in median glucose levels in the non-fasting (NF) group compared to the intermittent fasting (IF) group after cycle 4. **B** shows a significant increase in median insulin levels in the NF group compared to the IF group after cycle 4. Information is displayed by the box-and-whisker plot. **P < 0.01 and ***P < 0.001 vs. the other group

**Fig. 3:**
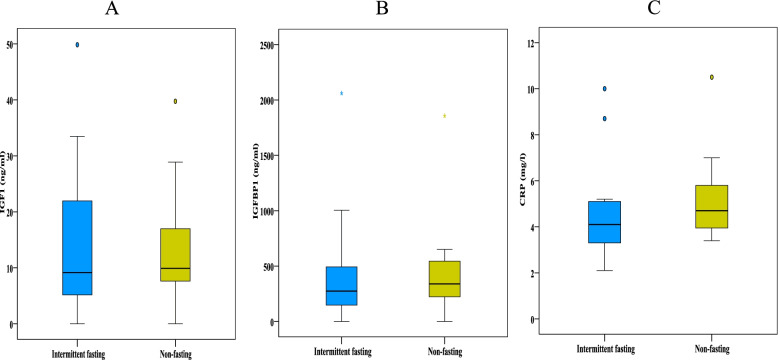
A comparison between the two studied groups according to IGF1 (ng/ml), IGFBP1 (ng/ml), and CRP (mg/l) after cycle 4. **A** shows no significant differences in IGF1 between the two studied groups after cycle 4. **B** shows no significant differences in IGFBP1 between the two studied groups after cycle 4. **C** shows no significant differences in CRP levels between the two studied groups after cycle 4. Information is displayed by the box-and-whisker plot. IGF1, insulin-like growth factor-1; IGFBP1, IGF-binding protein 1; CRP, C-reactive protein

There was a significant elevation of median glucose levels in the NF group compared to the IF group after cycle 4 (*P* < 0.001, 95% *CI* 23.39–60.49). Also, there was a significant elevation of median insulin levels in the NF group compared to the IF group after cycle 4 (*P* = 0.008, 95% *CI* 29.26–115.08) as shown in Fig. 2, while there were no significant differences in IGF1, IGFBP1, or CRP levels between the two studied groups after cycle 4 as shown in Fig. 3.

## Discussion

Preclinical studies prove that short-term fasting (STF) secures healthy cells from the side effects of chemotherapy while making cancer cells more vulnerable to it [9]. There has been an interest in investigating the effects of calorie intake and the nutritional composition of the diet on the chance of disease progression or recurrence in patients with cancer diagnoses [13]. STF has been studied to reduce chemotherapy side effects in cancer patients in limited clinical trials [11, 19].

This study was designed to explore the effects of IF on chemotherapy-induced side effects and to evaluate the ability of IF to promote DSR according to the metabolic changes induced in BC patients.

In our study, analysis of hematologic parameters showed no significant differences between the two groups until cycle 4, which showed no significant variations in chemotherapy-related modifications between the two groups.

Our results were in agreement with de Groot et al. [19], who randomized seven women with HER2-negative BC to fast for 24 h before their chemotherapy regimen of docetaxel, doxorubicin, and cyclophosphamide, and for 24 h after chemotherapy, significant elevation in platelet counts was observed in the STF group, explained by the decreased breakdown of circulating cells and/or less severe bone marrow suppression, but was incompatible with our results concerning erythrocyte counts, which showed significant elevation in the fasting group, explained by the hypothesis that STF may protect against chemotherapy-associated hematological toxicity.

In addition, our results showed no significant differences in median glucose levels in the IF group between baseline and after cycle 4, while there were significant increases in median glucose and median insulin levels in the NF group between the two points. In addition, there were significant increases in median glucose and in median insulin levels in the NF group compared to the IF group after cycle 4, which demonstrates the effect of IF throughout the day for 18 h in 3 consecutive days with the limitation of sugar and fats on the significant reduction of glucose and insulin levels, which may be due to better insulin performance and glucose tolerance [20, 21], despite the administration of dexamethasone as premedication.

In the NF group, the increase in glucose levels may lead to insulin elevation, which may be due to bad insulin performance and glucose intolerance [22]. The significant reduction in insulin levels in the IF group and the significant increase in glucose and insulin levels in the NF group may reflect the ability of IF in this way to promote DSR in BC patients despite the insignificant reduction in median IGF-1 levels in the IF group [10, 23–25].

DSR is said to occur when fasting limits the amount of glucose and insulin, among other hormones and metabolites, circulating in the body [10, 25].

Results obtained by de Groot et al. [19] were in disagreement with our results concerning plasma glucose levels, which increased in the STF group, which was explained by the use of dexamethasone.

Consistently with our results, Dorff et al. [11] showed that blood glucose levels did not change significantly or reliably among the compliant patients (*P* = 0.35), where three cohorts fasted for 24, 48, and 72 h before chemotherapy (48 pre-chemo and 24 post-chemo) and recorded all calories ingested. A total of 3/6 participants in each group consumed 200 kcal per 24 h during the fast phase without experiencing any adverse effects. In the 24-h cohort, there were four of six subjects stated fasting enforcement was registered in 200 kcal consumed; insulin levels were decreased in four of those patients by an average of 56% after chemotherapy after completing the first fast. Six compliant participants were included in the 48-h cohort, insulin levels in those patients decreased by 27%, and insulin levels in the seven compliant patients in the 72-h cohort had decreased by 42%.

In addition, Ferroni et al. [26] and Monzavi-Karbassi et al. [27] suggested in their studies in nondiabetic BC patients that elevated blood glucose and insulin levels were associated with a poor prognosis of breast cancer.

Lutes et al. [25] stated in their review concerning IF during chemotherapy that neither Dorff et al. [11] nor de Groot et al. [19] were able to show decreased levels of insulin and glucose when analyzing biochemical blood work.

The obvious findings of our study were that IF was well-tolerated, safe, and had a beneficial effect on patient-reported side effects, which may reflect the probability of promotion of DSR observed in the significant reduction in nausea, vomiting, diarrhea, and mouth sores, which may be due to exertion of some protection to gastrointestinal tract (GIT) cells against chemotherapy [8, 28].

Results obtained by Safdie et al. [8] in their case series study on ten patients suffering from different types of cancer were in agreement with our results concerning nausea, vomiting, diarrhea, and mouth sores which were virtually absent from the records of all ten patients in the fasting cycles which were undertaken prior to and/or following chemotherapy.

Our results demonstrated a significant increase in median CRP levels in NF patients. The lack of protection of GIT cells in the NF group may explain the significant increase in median CRP levels, and the presence of this protection in the IF group may explain the unchanged levels of median CRP in this group [29].

Poor glycemic control in the NF group may induce metabolic dysregulation, leading to pro-inflammatory conditions. Also, chronic inflammation may cause oxidative stress in the impaired glucose environment, which may accelerate tumor progression [22].

Our results showed a higher incidence of constipation in IF patients compared to NF patients. This obvious side effect related to IF in this way, which was constipation, may be due to dietary factors such as insufficient fiber intake or may be due to lifestyle factors such as lack of mobility or sedentary lifestyle [30].

Our results support the role of IF as a new intervention for malignancy treatment that can be utilized as an assistant to traditional treatment modalities, such as chemotherapy, to improve disease control.

Limitations of this study were the possibility of incomplete adherence to exact hours of fasting and the composition of the food consumed during the eating period, despite our encouragement to study subjects to honestly follow instructions about fasting hours and the food consumed regarding the total amount of calories consumed per day.

Examination of medication targets dependent on IGF-1 and IGFBP1 that might be similar to fasting in providing DSR will be a critical zone for future investigation.

## Conclusion

Intermittent fasting through the day in three consecutive days with a limitation of sugar and fat with a total amount of calories not exceeding 750 kcal per day is safe, feasible, and somewhat able to protect cancer patients from some chemotherapeutic toxicity related to GIT, which encourages the patient to adhere to chemotherapy treatment. IF improves the metabolic profiles of patients, which may affect the clinical viability of chemotherapy.

## Supplementary Information

**Additional file 1: Supplement 1.** The diet followed during the fasting days by the fasting group patients.

## Data Availability

The manuscript or additional files include all relevant materials.

## Abbreviations

IF: Intermittent fasting
AC: Doxorubicin and cyclophosphamide
BC: Breast cancer
HER2: Human epidermal growth factor receptor 2
NF: Non-fasting
GIT: Gastrointestinal tract
DSR: Differential stress resistance
IGF-1: Insulin-like growth factor-1
IGFBP-1: IGF-binding protein 1
LVEF: Left ventricular ejection fraction
LLN: Lower limit of normal
UNL: Upper normal limit
ALT: Alanine aminotransferase
AST: Aspartate aminotransferase
BMI: Body mass index
CRP: C-reactive protein
rpm: Round per minute
ELISA: Enzyme-linked immunosorbent assay
STF: Short-term fasting
