# Supplementary material for: Intermittent fasting during adjuvant chemotherapy may promote differential stress resistance in breast cancer patients
Source: J Egypt Natl Canc Inst. 2022 Sep 12;34:38. doi: 10.1186/s43046-022-00141-4 (PMC13314233; doi:10.1186/s43046-022-00141-4)
Supplement: Supplementary file 1 — Additional file 1: Supplement 1. The diet followed during the fasting days by the fasting group patients. [file 43046_2022_141_MOESM1_ESM.docx]

**Supplement 1: The diet followed during the fasting days by the fasting group patients**

| Time | First day | Second day | Third day | Total calories (Kcal) |
| --- | --- | --- | --- | --- |
| 11.00 pm | 1/4 loaf of bread +  4 tablespoons of beans without oil +  A cup of fat-free yogurt | 1/4 loaf of bread +  boiled egg  +  Cucumber slices | 1/4 loaf of bread + 4 tablespoons of beans without oil + A cup of fat-free yogurt | 100  110  60 |
| 6.00 pm | 3 dates  + A cup of fresh fruit juice without sugar | 3 dates  + A cup of fresh fruit juice without sugar | 3 dates  + A cup of fresh fruit juice without sugar | 60  25 |
| 6.30 pm | Boiled vegetable soup dish +  boiled red meat steak without fat  + 4 tablespoons of boiled rice or boiled pasta | Boiled vegetable soup dish +  one slice of boiled chicken without the skin + 4 tablespoons of boiled rice | salad plate  +  Grilled or boiled fish  +  4 spoons of boiled rice | 105  120  120 |
| 9.00 pm  (optional) | A piece of fruit (apple or guava) | A piece of fruit (apple or guava) | A piece of fruit (apple or guava) | 100 |
| Total calories (Kcal) |  |  |  | 700-800 |
